# Poly-γ-Glutamic Acid Induces Apoptosis via Reduction of COX-2 Expression in TPA-Induced HT-29 Human Colorectal Cancer Cells

**DOI:** 10.3390/ijms16047577

**Published:** 2015-04-03

**Authors:** Eun Ju Shin, Mi Jeong Sung, Jae Ho Park, Hye Jeong Yang, Myung Sunny Kim, Haeng Jeon Hur, Jin-Taek Hwang

**Affiliations:** 1 Department of Food Biotechnology, University of Science & Technology, 217 Gajeong-ro, Yuseong-gu, Daejeon 305-333, Korea; E-Mails: sej296@hanmail.net (E.J.S.); dulle5@kfri.re.kr (M.J.S.); jhpark@kfri.re.kr (J.H.P.); 2 Korea Food Research Institute, 1201 Anyangpangyoro, Bundang-gu, Seongnam-si, Gyeonggi-do 463-746, Korea; E-Mails: yhj@kfri.re.kr (H.J.Y.); truka@kfri.re.kr (M.S.K.); mismalto@kfri.re.kr (H.J.H.)

**Keywords:** poly-γ-glutamic acid, HT-29 human colorectal cancer cell, apoptosis, cyclooxygenase, AMP-activated protein kinase

## Abstract

Poly-γ-glutamic acid (PGA) is one of the bioactive compounds found in *cheonggukjang*, a fast-fermented soybean paste widely utilized in Korean cooking. PGA is reported to have a number of beneficial health effects, and interestingly, it has been identified as a possible anti-cancer compound through its ability to promote apoptosis in cancer cells, although the precise molecular mechanisms remain unclear. Our findings demonstrate that PGA inhibits the pro-proliferative functions of the phorbol ester 12-*O*-tetradecanoylphorbol-13-acetate (TPA), a known chemical carcinogen in HT-29 human colorectal cancer cells. This inhibition was accompanied by hallmark apoptotic phenotypes, including DNA fragmentation and the cleavage of poly (ADP-ribose) polymerase (PARP) and caspase 3. In addition, PGA treatment reduced the expression of genes known to be overexpressed in colorectal cancer cells, including cyclooxygenase 2 (COX-2) and inducible nitric oxide synthase (iNOS). Lastly, PGA promoted activation of 5' adenosine monophosphate-activated protein (AMPK) in HT-29 cells. Taken together, our results suggest that PGA treatment enhances apoptosis in colorectal cancer cells, in part by modulating the activity of the COX-2 and AMPK signaling pathways. These anti-cancer functions of PGA make it a promising compound for future study.

## 1. Introduction

Cancer cells are characterized by deregulation of the intrinsic signaling mechanisms responsible for cellular growth, proliferation, and survival. Cell and tissue homeostasis depends heavily on a critical balance of these opposing processes, meaning that their control is vital toward maintaining a healthy, normally functioning cell [1]. Colorectal cancer is the third deadliest cancer in the United States and it has recently become much more prevalent in Asia [2]. The standard of care for colorectal cancer is currently surgical resection and chemotherapy, although there is significant interest in treatments involving naturally occurring compounds that may reverse, suppress, or prevent the development of epithelial malignancies. The efficacy and mechanism of action of these natural remedies has been the subject of recent study.

The focus of several labs has been, in particular, the ability of these compounds to promote apoptosis in transformed cells by shifting the balance of apoptotic regulatory proteins [3]. Cyclooxygenase 2 (COX-2), a well-known pro-survival signaling molecule, is present at elevated levels in colorectal cancer [4]. Our previous work revealed that inhibition of COX-2 by selenium treatment induces apoptosis in HT-29 colorectal cancer cells [5]. We found that COX-2 inhibition by (−) epigallocatechin-3-gallate (EGCG), a green tea polyphenol, also induces apoptosis [6]. Together, these findings demonstrated that COX-2 is a significant pro-survival protein in colorectal cancer cells, and that inhibition of COX-2 is an effective option for colorectal cancer intervention [5,6,7,8]. In addition to our work in colorectal cancer lines, a number of authors have stressed the importance of COX-2 signaling for abnormal cell survival across a variety of cancer types [5,6,7,8]. Importantly, the apoptosis-inducing treatments of either green tea polyphenol or selenium also resulted in activation of AMP kinase (AMPK), a known signaling molecule upstream of cellular apoptotic functions. AMPK plays a central role in maintaining the balance between anabolism and catabolism under conditions of cellular stress, such as energy deprivation and hypoxia [9]. AMPK agonists, such as metformin, AICAR, and other naturally derived compounds, have been shown to be effective at inducing apoptosis in cancer cells [9,10,11,12,13].

Poly-γ-glutamic acid (PGA) is an anionic polymer composed of d- and l-glutamic acid units connected by γ-amide linkages between alpha-amino and γ-carboxylic acid groups [14]. It is produced as a byproduct of fermentation from *Bacillus subtilis* and is found in *cheonggukjang*, a Korean fermented soybean paste, which has been shown to provide beneficial health effects in certain disease models [14]. PGA is water-soluble, biodegradable, edible, and non-toxic. Previous studies have identified potential anti-tumorigenic, anti-angiogenic, and anti-inflammatory properties of PGA, although the mechanisms by which it promotes these effects are unclear [15,16]. The purpose of this work was to identify the effector pathways responsible for the anti-cancer properties of PGA, specifically, in the HT-29 human colorectal cancer cell line. We determined that PGA induces canonical apoptotic cell death marked by decreased COX-2 expression while also promoting AMPK activation.

## 2. Results

### 2.1. Treatment with Poly-γ-Glutamic Acid (PGA) Has an Anti-Proliferative Effect on HT-29

**Figure 1:**
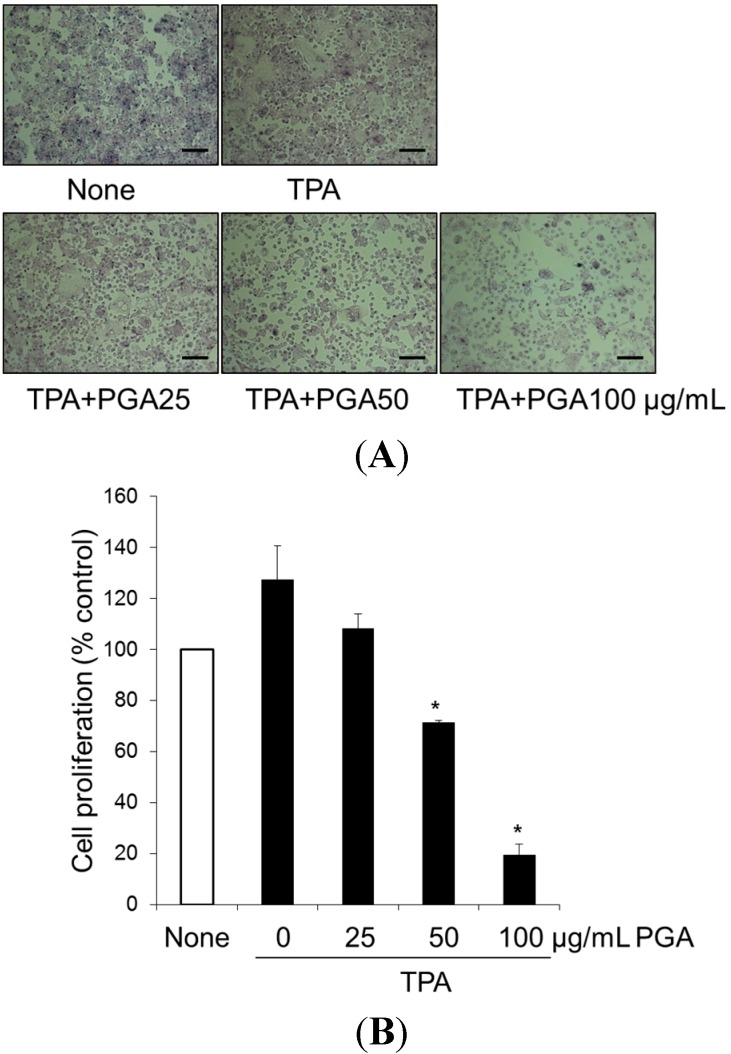
Effect of poly-γ-glutamic acid (PGA) on TPA-induced proliferation in HT-29 human colorectal cancer cells. Cells were treated with 100 nM 12-*O*-tetradecanoylphorbol-13-acetate (TPA), alone or in combination with PGA, at 25, 50, 100 μg/mL for 48 h, and then morphological changes of HT-29 cells were observed under phase-contrast microscope (**A**). Scale bar = 20 µm; Under same condition, cell proliferation was measured using the (4,5-dimethylthiazol-2-yl)-2,5 diphenyltetrazolium bromide (MTT) assay (**B**). Data representative of three independent experiments and were expressed as mean ± SD. * *p* < 0.05 *vs.* TPA alone.

Previous studies have demonstrated that 12-*O*-tetradecanoylphorbol-13-acetate (TPA), a carcinogenic agent stimulates cancer cell proliferation via induction of growth-related proteins, including COX-2 in cancer cells [17,18,19]. Thus, we first investigated whether PGA can inhibit TPA-induced HT-29 cell proliferation. To this end, cells were treated with TPA alone or in combination with PGA (25, 50, 100 µg/mL) for 48 h. As shown in Figure 1A,B, TPA alone enhanced cellular proliferation by about 30%. In contrast, TPA combined with PGA showed morphological changes, including cell shrinkage (Figure 1A) and inhibited HT-29 cell proliferation (Figure 1B). These results identified PGA as an effective inhibitor of TPA-stimulated proliferation in HT-29 cells.

### 2.2. PGA Induces Apoptosis in HT-29 Cells

**Figure 2:**
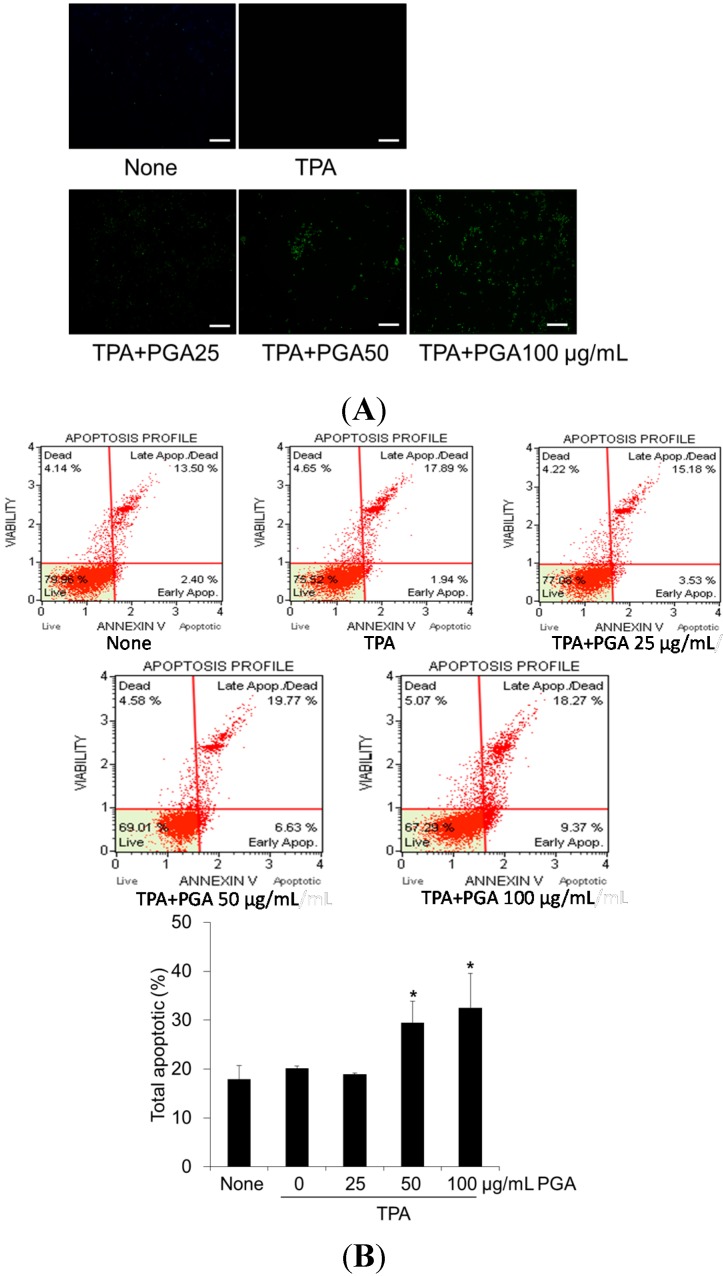

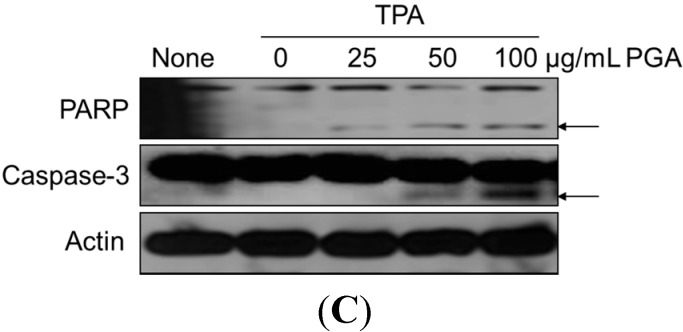
Effect of PGA on apoptosis in TPA-induced HT-29 cells. Cells were treated with 100 nM 12-*O*-tetradecanoylphorbol-13-acetate (TPA), alone or in combination with PGA, at 25, 50, 100 μg/mL for 48 h, and then apoptosis was measured by TUNEL assay (**A**) Scale bar = 20 µm; Under the same condition, apoptosis was also measured by Annexin V staining, and quantitative analysis of the percentage of total apoptotic cells was represented by graphs (**B**); Cells were treated with 12-*O*-tetradecanoylphorbol-13-acetate (TPA), and different concentrations of PGA at 25, 50, 100 μg/mL for 24 h, and then PARP and caspase-3 were measured by Western blot analysis (**C**). Data are expressed as mean ± SD. * *p* < 0.05 *vs.* TPA alone.

To determine whether the inhibitory effects of PGA on HT-29 cellular proliferation are accompanied by enhanced apoptosis, we utilized the terminal deoxynucleotidyl transferase dUTP nick end labeling (TUNEL) assay to examine DNA fragmentation, a common identifier of the final stage of apoptotic cell death. As shown in Figure 2A, cells treated with PGA displayed a marked increase in TUNEL staining, implicating PGA-dependent apoptotic cell death occurring in a dose-dependent manner. To confirm the TUNEL results, we next utilized Annexin V staining to detect phosphatidyl serine membrane translocation, another hallmark of late-stage apoptosis. As shown in Figure 2B, treatment with PGA resulted in a higher percentage of apoptotic cells when compared to control treatments. Finally, we examined the cleavage states of well-studied pro-apoptotic proteins in response to PGA treatment. When compared to control treatments, PGA resulted in significant cleavage of both PARP and caspase-3 (Figure 2C). All together, these results suggest that PGA treatment induces apoptosis in HT-29 cells.

**Figure 3:**
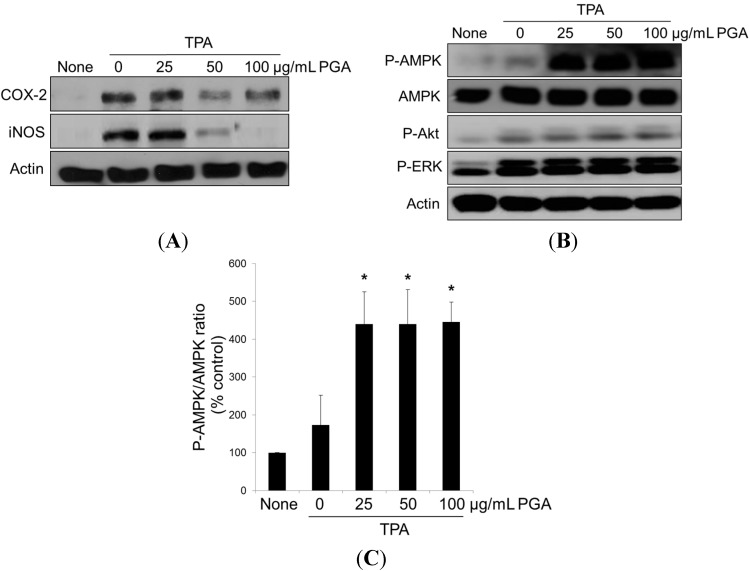
Effect of PGA on COX-2, iNOS, and AMPK in TPA-induced HT-29 cells. Cells were treated with 100 nM 12-*O*-tetradecanoylphorbol-13-acetate (TPA), alone or in combination with PGA, at 25, 50, 100 μg/mL for 24 h, and then COX-2 and iNOS expressions were determined under a Western blot analysis (**A**); Cells were treated with 12-*O*-tetradecanoylphorbol-13-acetate (TPA), and different concentrations of PGA at 25, 50, 100 μg/mL for 1 h, and then AMPK, Akt, and ERK phosphorylations were determined by Western blot analysis with specific antibodies (**B**); and each bar represents the P-AMPK/AMPK protein ratio (**C**). * *p* < 0.05 *vs.* TPA alone

### 2.3. PGA Reduces TPA-Dependent COX-2 and Inducible Nitric Oxide Synthase (iNOS) Expression and Promotes AMPK Phosphorylation in HT-29 Cells

Overexpression of both iNOS and COX-2 has been reported in colorectal cancer [6,7,8]. Previous studies identified COX-2 as an efficacious drug target that, when inhibited, promotes apoptosis in TPA-induced cancer cell lines [6,7,8]. Given the dramatic apoptotic response displayed following PGA treatment, we investigated whether PGA treatment reduces the TPA-dependent overexpression of COX-2 and iNOS in HT-29 cells. As shown in Figure 3A, treatment with TPA caused a predictable increase in COX-2 expression. Strikingly, combination treatment with both TPA and PGA almost reversed the TPA-induced overexpression of iNOS and COX-2.We next examined known apoptotic signaling cascades in an effort to determine the factors mediating PGA-dependent apoptosis. Cells receiving TPA alone displayed increased ERK phosphorylation, but this had little to no effect on AMPK and Akt. Interestingly, combination treatment with both TPA and PGA promoted AMPK phosphorylation, but this had no effect on ERK and Akt (Figure 3B,C). Taken together, these data suggest that both COX-2 and AMPK signaling pathways are potential targets of PGA leading to the induction of apoptotic cell death of colorectal cancer cells.

## 3. Discussion

Cancer is an inherently complicated disease, often characterized by an inability to maintain the necessary balance between growth and proliferation and cell death [1]. Such dysfunction may be triggered by a vast array of stimuli, including growth factors, cytokines, and environmental toxins or chemicals [1,20]. Recently, a number of studies have focused on the use of naturally occurring compounds and their possible utility as anti-cancer agents, particularly due to their ability to tip this delicate balance and promote apoptosis; A programmed form of cell death accompanied by DNA fragmentation, positive TUNEL/Annexin V staining, and cleavage of pro-apoptotic proteins [1,3,20]. Previously, we demonstrated that AMPK activation through treatment with selenium or EGCG negatively regulates COX-2 in colorectal cancer cells [5,6].

The purpose of this study was to characterize the anti-cancer properties of another naturally occurring compound, PGA, in the HT-29 human colorectal cancer cell line. Because TPA is a well-known carcinogen [17,18,19], HT-29 cells were treated with TPA alone or in combination with PGA. TPA alone increased cellular proliferation by about 30%. Combination of PGA and TPA has anti-proliferative, as well as pro-apoptotic functions in HT-29 cells (Figure 1 and Figure 2). In agreement, we found that apoptotic proteins such as PARP and caspase 3 were significantly cleaved in response to PGA treatment in the TPA-treated HT-29 cells (Figure 2C), further demonstrating that PGA promotes apoptosis. On the other hand, PGA treatment alone had only 10% reduction of HT-29 cell proliferation (data not shown). These results demonstrate that PGA exhibits strong anti-proliferative effect against carcinogen-stimulated colorectal cancer cells.

We also identified a mechanism through which PGA-dependent cell death occurs, involving changes in COX-2 expression and AMPK activity. But this had no effect on ERK and Akt. PI3-kinase/Akt signaling pathway plays a central role in tumor progression [21,22]. Several anti-cancer agents and bioactive compounds have been demonstrated that effectively inhibits cancer cell growth by targeting Akt signaling pathways [21,22]. It is reported that reduced Akt activity increases mitochondrial Bad, thereby leading to the mitochondria-dependent apoptosis [23]. Therefore, we speculate that PI3K-Akt-Bad signaling pathways may not be involved in PGA-inhibited cancer cell growth. On the other hand, COX-2 is a pro-survival signaling molecule, and several naturally occurring dietary agents have been previously identified as inhibitors of its expression [4,5,6]. Such compounds include luteolin, apigenin, genistein, wogonin, green tea catechins, curcumin, and resveratrol [24,25,26,27,28,29,30]. Many of these compounds also activate AMPK in various cancer cells, resulting in induction of apoptosis [24,25,26,27,28,29,30]. In this study, we identified PGA as another naturally occurring dietary compound that can reduce expression of COX-2 and iNOS and promote apoptosis. The mechanism outlined above is in agreement with previous work in berberine-treated colorectal cancer cells [31]. Berberine activates AMPK and inhibits mTOR and COX-2 signaling pathways, resulting in inhibition of proliferation [31].

Although we have identified the importance of AMPK and COX-2 signaling pathways in PGA-dependent apoptotic programs, we have not addressed how PGA regulates the activity of the AMPK and COX-2 signaling pathways, or how it contributes to the cleavage of pro-apoptotic factors such as PARP and caspase-3. Further studies are needed to answer these questions. Altogether, these results provide further evidence that PGA has the potential to promote apoptosis in colorectal cancer cells, providing vital understanding toward the development of targeted molecular therapies.

## 4. Material and Method

### 4.1. Cell Culture Conditions and Reagents

The HT-29 human colorectal cancer cell line was purchased from American Type Culture Collection (Manassas, VA, USA). Cells were cultured in Roswell Park Memorial Institute medium (RPMI) 1640 medium supplemented with 10% fetal bovine serum (WelGene, Daegu, Korea) and 100 µg/mL streptomycin (WelGene) at 37 °C in a 5% CO_2_ atmosphere. RPMI was purchased from WelGene, PGA was purchased from Bioleaders (Daejeon, Korea), and 12-*O*-tetradecanoylphorbol-13-acetate (TPA) was purchased from Sigma (St. Louis, MO, USA). TUNEL and Annexin V assay kits were purchased from Millipore (Darmstadt, Germany). Antibodies against phospho-AMPK (Thr172), phospho-ERK (Thr202/Tyr204), phospho-Akt (Thr308), caspase 3, and AMPK from Cell Signaling Technology (Beverly, MA, USA). COX-2 and iNOS antibodies were purchased from Cayman (Ann Arbor, MI, USA). PARP antibody was purchased from Santa Cruz Biotechnology (Santa Cruz, CA, USA). β-Actin antibody was purchased from Bethyl Laboratories (Montgomery, TX, USA).

### 4.2. Cell Proliferation Assay

Cell proliferation was measured by the (4,5-dimethylthiazol-2-yl)-2,5 diphenyltetrazolium bromide (MTT) assay. Cells seeded on 24-well microplates at 5 × 10^4^ cells/well were treated with PGA for 48 h and exposed to 10 μL of MTT solution (5 mg/mL MTT in phosphate-buffered saline (PBS)) for 3 h. Live cells appeared purple in color in response to MTT. After discarding the medium, cells were dissolved in dimethyl sulfoxide. One hundred microliters of cell suspension was transferred to a 96-well plate and cell viability was determined using VersaMax™ microplate ELISA reader (Molecular Device Co., Sunnyvale, CA, USA).

### 4.3. Annexin V Assay

Cells were co-treated with vehicle or TPA and 25, 50, 100 μg/mL PGA for 48 h. Following treatment, cells were collected, centrifuged at 12,000 rpm for 2 min, and resuspended in 100 µL of PBS. One hundred microliters of MUSE Annexin V reagent was added to the cellular suspensions before incubating for 20 min in the dark room. Apoptotic cells were identified using a MUSE cell analyzer (Millipore).

### 4.4. TUNEL Assay

Cells were co-treated with vehicle or TPA and 25, 50, 100 μg/mL PGA for 48 h. The TUNEL assay kit was used as previously described [32]. DNA fragmentation was detected by fluorescence microscopy (Olympus, Tokyo, Japan).

### 4.5. Western Blot Analysis

Following treatment with TPA, PGA, or both, crude HT-29 cell extracts were prepared in lysis buffer (Elpis; Daejeon, Korea) supplemented with protease and phosphatase inhibitor cocktails (Roche; Basel, Switzerland). Proteins were resolved on 10% Bis-Tris gels, and then transferred to a polyvinyl difluoride membrane. The membrane was blocked in 5% blocking buffer for 1 h and probed with specific antibodies overnight as noted in the figures. Membranes were incubated with horseradish peroxidase-conjugated secondary antibodies (Enzo Life Sciences; Farmingdale, NY, USA) and the chemiluminescence method was used for detection.

### 4.6. Statistical Analysis

Data are presented as means ± standard deviation (SD) and were analyzed by nonparametric methods using the SPSS computer-based statistics programs (Ver. 20; SPSS Inc., Chicago, IL, USA). Statistical differences between means were evaluated by one-way analysis of variance (ANOVA) followed by Bonferroni test. The value of *p* < 0.05 was considered to be significant.
